# Acupuncture Treatment for Social Defeat Stress

**DOI:** 10.3389/fnbeh.2021.685433

**Published:** 2021-07-28

**Authors:** Jun Kawanokuchi, Ken Takagi, Nobuyuki Tanahashi, Teruhisa Yamamoto, Nobuyuki Nagaoka, Torao Ishida, Ning Ma

**Affiliations:** ^1^Institute of Traditional Chinese Medicine, Suzuka University of Medical Science, Suzuka, Japan; ^2^Division of Health Science, Graduate School of Health Science, Suzuka University of Medical Science, Suzuka, Japan; ^3^Department of Acupuncture and Moxibustion Science, Faculty of Health Science, Suzuka University of Medical Science, Suzuka, Japan; ^4^Department of Clinical Nutrition, Faculty of Health Science, Suzuka University of Medical Science, Suzuka, Japan

**Keywords:** depression, social defeat stress, acupuncture, neurotrophic factors, BDNF, NT-3

## Abstract

Depression is a mood disorder characterized by disordered affect, thoughts, cognition, and behavior. Antidepressant therapy is often the primary treatment for depression. However, antidepressant therapy may cause unwanted side effects, and its effects are slow. Therefore, some patients are seeking alternative treatments for depression, such as acupuncture. However, there are many unclear points regarding the mechanism of the effect of acupuncture on depression. In recent years, we have reported that acupuncture improves the symptoms of mild depression induced by water-immersion stress in a rat model and depression induced by forced swimming in a mouse model. In this study, we examined the effect of acupuncture on the symptoms of social defeat stress (SDS)-induced depression in mice that most closely resemble human symptoms. In this study, we investigated the preventive and therapeutic effects of acupuncture as part of GV20 “Bai-Hui” and Ex-HN3 “Yintang” on model mice with depression induced by SDS. To examine the mechanism of the preventive and therapeutic effects of acupuncture on depression model mice, we examined the expression of neurotrophic factors in the brains of SDS mice. Two weeks of simultaneous acupuncture stimulation as part of GV20 and Ex-HN3 restored SDS-reduced brain-derived neurotrophic factor (BDNF), neurotrophin (NT)-3, and NT-4/5 expression, which was not observed with antidepressants. In contrast, acupuncture stimulation suppressed nerve growth factor (NGF) expression induced by SDS. These results suggest that acupuncture treatment could be effective in correcting the imbalance in the expression of neurotrophic factors. Furthermore, the effects of acupuncture on the expression of neurotrophic factors appear earlier than those of antidepressants, suggesting that it may be a useful treatment for depression.

## Introduction

In recent years, the number of patients with psychiatric disorders has increased in many countries, including Japan. According to a survey conducted in 2017, depressive disorder is a common mental illness affecting more than 300 million people of all ages worldwide (WHO Fact Sheet 2017 on Depression) (WHO, 2017). Depression is a mood disorder characterized by disordered affect, thoughts, cognition, and behavior. Social loss due to depression is widespread as it may cause suicide and obstacles in work and school.

Depression is commonly treated with psychotherapy, antidepressant therapy (Kirsch et al., 2008), and/or electroconvulsive therapy (Schlaepfer et al., 2010). Antidepressant therapy is often the primary treatment for depression. However, antidepressant therapy may cause unwanted side effects, and its effects are slow (Khawam et al., 2006; Cipriani et al., 2018). In addition, about one-third of patients are known to have treatment-resistant depression to multiple antidepressants (Al-Harbi, 2012). Therefore, some patients are seeking alternative treatments for depression, such as acupuncture therapy (Qureshi and Al-Bedah, 2013).

Acupuncture is a traditional Chinese method widely used for treating a variety of physical and mental health problems. John Kim (2007) reviewed the biological mechanisms of acupuncture and the effectiveness of electroacupuncture stimulation on depression as part of GV20 “Bai-Hui” and Ex-HN3 “Yintang.” It has been reported that acupuncture at the acupoints GV20 and Ex-HN3 can induce sedation and provide relief from stress in animals and humans (Litscher, 2004; Paraskeva et al., 2004; Kim and Nam, 2006). A pilot study by Yeung et al. (2011) suggested that acupuncture is safe, well-tolerated, and effective for partial and non-responders to antidepressants. Discussions on the standardization of acupuncture points began in 2003, and the “WHO Standard Acupuncture Point Locations in the Western Pacific Region” (WHO Standard) containing these acupuncture points were released in 2008 (WHO, 2008). However, there are many unclear points regarding the mechanism of the effect of acupuncture on depression. Animal studies are commonly used in depression research, but few have investigated the effects of acupuncture on depression. Dos Santos et al. (2008) reported that depression model rats induced by forced swimming (FS) stimulated with electroacupuncture at the SP-36 “Zusanli” and Sp-6 “Sanyinjiao” points with electroacupuncture showed antidepressant effects comparable to those of the antidepressant imipramine. In addition, verification of the effect of electroacupuncture at the GV20 and Ex-HN3 points (Liu et al., 2007), GV20 and Sp-6 points (You et al., 2010), or GV20 and EX17 “An-Mian” We have reported that acupuncture, as part of GV20 and Ex-HN3, improves the symptoms of a rat model of mild depression induced by water-immersion stress (Tanahashi et al., 2016; Takagi et al., 2017) and a mouse model of depression induced by FS (Yamamoto et al., 2018). Social defeat stress (SDS) in mice has been developed as a new ethological model of depression that more closely models the psychological stress that humans can experience during antagonistic social interactions (Golden et al., 2011; Toyoda, 2017). Since the SDS-induced depression model is caused by mental stress due to competition and defeat between individuals, it is considered that the SDS model reproduces human depression more than the previous animal models. In this study, we examined the effects of acupuncture on the suppression of depressive-like symptoms in an SDS-induced depression mouse model that is attacked by other animals to mimic the mental stress response that would occur in a more natural environment. Depression-like symptoms were assessed by measuring changes in immobility time using the forced swimming test (FST), which is used as a screening test for antidepressants. Acupuncture was performed on a pair of acupoints of GV20 and EX-HN3 of SDS mice. GV20 is located at inferior border of the occipital protuberance on the vertical midline of posterior of the head. Ex-HN3 is located at the midpoint of glabella between the inner/medial ends of the eyebrows on the face. The classic tricyclic antidepressant, imipramine, was used as a positive control for treatment.

Many studies have been conducted on the mechanisms of depression development in humans and animals. One of the leading hypotheses is the “Neurotrophic factor hypothesis,” which explains that the decreased expression of neurotrophic factor, such as a brain-derived neurotrophic factor (BDNF), is related to the development of depression. Neurotrophic factors are composed of four types of secretory proteins: nerve growth factor (NGF), BDNF, neurotrophin-3 (NT-3), and neurotrophin-4/5 (NT-4/5). Focusing on these neurotrophic factors, we investigated the therapeutic mechanism of acupuncture in SDS-induced depression model mice.

## Materials and Methods

### Experimental Animals

All protocols were approved by the Animal Experiment Ethical Review Committee of the Suzuka University of Medical Science (permission number: 189). 6-week-old ICR male mice and C57BL/6J male mice were purchased from CLEA Japan, Inc. (Tokyo, Japan). Animals were maintained at a room temperature of 22 ± 3°C, 55 ± 5% humidity, and a 12-h light/dark cycle. Mice were given free access to commercially available animal feed (CE-2; CLEA Japan, Inc.) and water. Seven-week-old C57BL/6J mice were acclimatized to handling for 1 week before the start of the experiment. In this study, male mice were used to eliminate the effects of the estrous cycle. The number of animals used in the experiment was set to a minimum of eight animals per group. C57BL/6J mice (*n* = 64) in eight groups and 48 ICR mice (*n* = 48) in six groups were used. No mice were excluded from the experiment due to death or abnormalities.

### Production of Depressive-Like Symptom in Mice

A depressive-like symptom (increased immobility) was induced in C57BL/6J mice using the SDS method (according to the method of Golden) (Golden et al., 2011). An ICR mouse was housed in a compartment separated by a transparent acrylic divider containing many holes. After a few days of habituation, a C57BL/6J mouse is introduced into this compartment, and the ICR mouse would usually severely attack the C57BL/6J mouse. After several minutes of this social conflict, the C57BL/6J mouse was moved to an adjacent compartment for the remainder of the day. The following day, the C57BL/6J mouse was subjected to social conflict with another ICR mouse. This sequence of physical and psychological stress was repeated for 14 days to increase SDS-induced immobility in C57BL/6J mice. The weight of each mouse was measured weekly.

### Acupuncture or Pharmacotherapy in SDS-Treated Mice

A depressive-like symptom (increased immobility) was induced in mice with 2 weeks of SDS. Acupuncture stimulations or imipramine administration were provided as antidepressant therapies for increased immobility of SDS-treated mice. The classic tricyclic antidepressant imipramine was used as a positive control.

**FIGURE 1:**
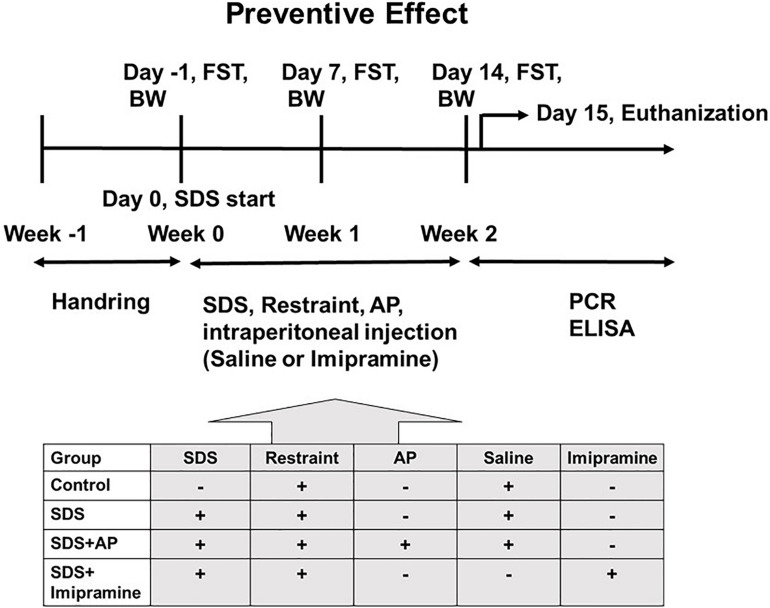
The schedule of the “preventive effect” experiment. The timeline of the procedures for the “preventive” group conditions. AP, acupuncture; BW, body weight; ELISA: enzyme-linked immuno sorbent assay; FST, forced swimming test; PCR, polymerase chain reaction; SDS, social defeat stress.

**FIGURE 2:**
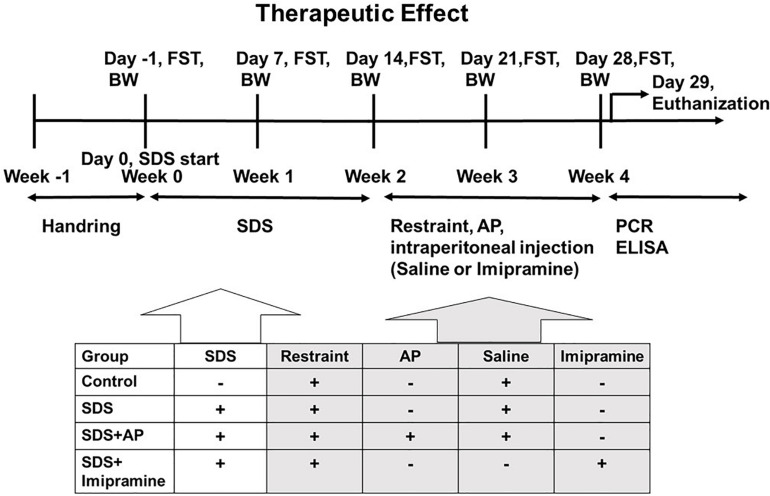
The schedule of the “therapeutic effect” experiment. The timeline of the procedures for the “therapeutic” group conditions. AP, acupuncture; BW, body weight; ELISA, enzyme-linked immuno sorbent assay; FST, forced swimming test; PCR, polymerase chain reaction; SDS, social defeat stress.

First, the animals were divided into two groups. The first group was an experimental group that examined the effect of acupuncture in preventing the increased immobility of SDS-treated mice, and the treatments were conducted during the preparation of the SDS-treated mice (Figure 1). The second group was an experimental group that examined the therapeutic effect of acupuncture on the already developed increased immobility, and the treatments were performed after the SDS-treating period (Figure 2).

These two groups of mice were each randomly divided into four experimental groups (*n* = 8 per group): (1) Control group: SDS (–), tail restraint (+), saline (+); (2) SDS group: SDS (+), tail restraint (+), saline (+); (3) SDS + Acupuncture group: SDS (+), tail restraint (+), acupuncture (GV20 and Ex-HN3), saline (+); and (4) SDS + Imipramine group: SDS (+), tail restraint (+), and imipramine (+).

#### Acupuncture Stimulation

**FIGURE 3:**
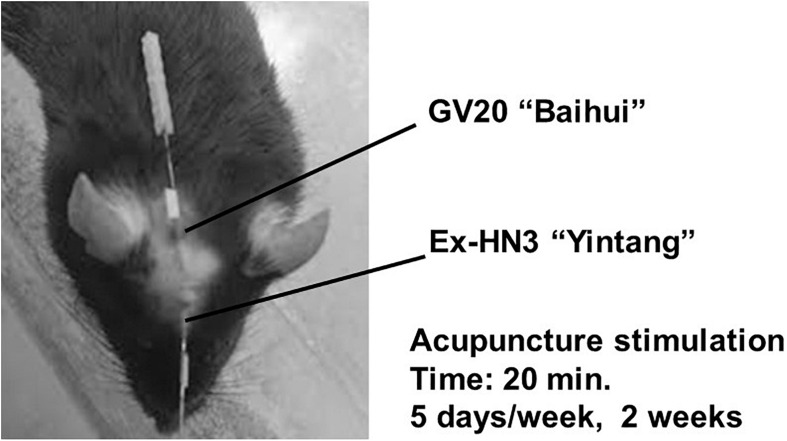
Acupuncture points. Horizontal and inward simultaneous acupuncture stimuli were continued for 20 min with insertion to a depth of 5 mm at the “Bai-Hui” (GV20) and “Yintáng” (Ex-HN3) acupuncture points using stainless steel needles (diameter, 0.25 mm).

In brief, simultaneous horizontal and inward acupuncture stimuli were continuously given for 20 min with insertion to a depth of 5 mm at the “Bai-Hui” (GV20) and “Yintáng” (Ex-HN3) acupuncture points of mice using stainless steel needles (Acupuncture Needle D-Type, diameter: 0.25 mm; length: 15 mm; SEIRIN Co., Ltd., Shizuoka, Japan). These stimulation points and penetration depths were anatomically analogous to those in rats (Figure 3). The method of acupuncture stimulation was based on a previous report (Tanahashi et al., 2016; Takagi et al., 2017; Yamamoto et al., 2018). During acupuncture stimulation, the mouse was fixed with adhesive tape on its tail in an overturned cage. These acupuncture stimuli were performed 10 days out of 2 weeks (20 min daily, Monday through Friday). Groups that did not receive acupuncture stimulation were fixed at the same time as the acupuncture group. All acupuncture treatments and fixations were performed between 10:00 and 14:00. The time of day that acupuncture was performed and the frequency of acupuncture were similar in the “preventive” and “therapeutic” groups. It usually takes 4 weeks for imipramine to work, but our previous studies have shown that imipramine can work in 2 weeks while depressive-like symptoms are increasing (Tanahashi et al., 2016).

#### Administration of Antidepressant

The dose of antidepressants was set according to previous reports (Porsolt et al., 1977; Adams et al., 2008; Tanahashi et al., 2016). Imipramine (10 mg/kg body weight; Sigma-Aldrich, St. Louis, MO, United States) was dissolved in saline and administered intraperitoneally 5 days/week for 2 weeks. Groups that did not receive pharmacotherapy were injected intraperitoneally with saline. Saline and imipramine injections were administered within 10 min after acupuncture or fixation.

### Forced Swimming Test

Forced Swimming Test was performed using the method described by Porsolt et al. (1977). Mice were placed in a plastic beaker (diameter 15 cm, height 22 cm) containing freshwater at 25 ± 2°C to a depth of body height of +5 cm for 6 min. The action of the mouse was recorded with a video camera, and the immobile time for 5 min after excluding the first 1 min from the 6-min recording time was measured. The immobile state is defined as a state in which the mouse suspends most movements and performs only the limb movements necessary to maintain balance. Brief swimming stops of less than 1 s in stopping time were not counted as immobile time. In all groups, FST was performed between 14:00 and 16:00.

### Sample Collection

Mice were euthanized by intraperitoneal injection of barbital sodium salt solution (120 mg/kg) 18***–***20 h after the last FST. Brains were immediately removed and stored at ***−***80°C, and the right anterior cerebrum was used for molecular biological and biochemical analysis.

### RNA Extraction and Reverse Transcription-Polymerase Chain Reaction for Neurotrophic Factors

Total RNA was extracted from brain tissue using the RNeasy Mini kit according to the manufacturer’s protocol (Qiagen, Valencia, CA, United States). The amount of RNA was determined spectrophotometrically. cDNAs encoding mouse GAPDH, NGF, BDNF, NT-3, and NT-4/5 were evaluated by reverse transcription-polymerase chain reaction (RT-PCR) using Super Script III (Invitrogen, Carlsbad, CA, United States) and Blend Taq DNA polymerase (Toyobo, Osaka, Japan) in the presence of the specific primers shown in Table 1. To quantify the expression, mRNA levels of neurotrophic factors were also analyzed by real-time RT-PCR using PowerUp SYBR Green Master Mix and ABI Prism 7,000 SDS according to the manufacturer’s protocol (Thermo Fisher Scientific, Waltham, MA, United States). The specific primers that were used are shown in Table 2. To prevent contamination of genomic DNA, primer sequences were set to amplify regions containing many introns. The Standard Curve Method was used as the quantitation method. A calibration curve was prepared for each reaction system, and the quantitative values were calculated.

**TABLE 1:** Primer sets for PCR analysis.

Molecule	Forward primer	Reverse primer
GAPDH	ACTCACGGGAAATTCAACG	CCCTGTTGCTGTAGCCGTA
NGF	CATGGGGGAGTTCTCAGTGT	GCACCCACTCTCAACAGGAT
BDNF	AGCCTCCTCTGCTCTTTCTG	TTGTCTATGCCCCTGCAGCC
NT-3	GCCTACGAGTTTGTTGTTTTC	ATGCAGAGCATAAGAGTCAC
NT-4/5	AGCCGGGGAGCAGAGAAG	ACAAGAGGTCCCACTCAGGA

*Primer sequences used for reverse transcription-polymerase chain reaction (RT-PCR) analysis. Annealing temperature and PCR cycles: GAPDH (58°C, 28 cycles), NGF (58°C, 35 cycles), BDNF (62°C, 30 cycles), NT-3 (62°C, 35 cycles), and NT-4/5 (65°C, 33 cycles).*

**TABLE 2:** Primer sets for real-time PCR.

Molecule	Forward primer	Reverse primer
GAPDH	ATGGGAGTTGCTGTTGAAGTCA	CCGAGGGCCCACTAAAGG
NGF	GATCGGCGTACAGGCAGAAC	CAGTGGGCTTCAGGGACAGA
BDNF	CCAAAGGCCAACTGAAGCAGTA	GCAGCCTTCCTTGGTGTAACC
NT-3	TTCTGCCACGATCTTACAGG	GGCAAACTCCTTTGATCCAT
NT-4/5	AGCGTTGCCTAGGAATACAGC	GGTCATGTTGGATGGGAGGTATC

*Annealing temperature and PCR cycles: (55°C, 45 cycles).*

### ELISA

The expression of cerebral neurotrophic factors was assessed using a Multi-Neurotrophin Rapid Screening ELISA kit (Mouse) according to the manufacturer’s protocol (Biosensis, SA, Australia).

### Statistical Analysis

Statistical significance was assessed using one-way analysis of variance with Tukey–Kramer *post hoc* test using PRISM (version 5.0; GraphPad Software, La Jolla, CA, United States). Statistical significance was set at *p* < 0.05.

## Results

### Physical and Behavioral Studies on the SDS-Treated Mice

**FIGURE 4:**
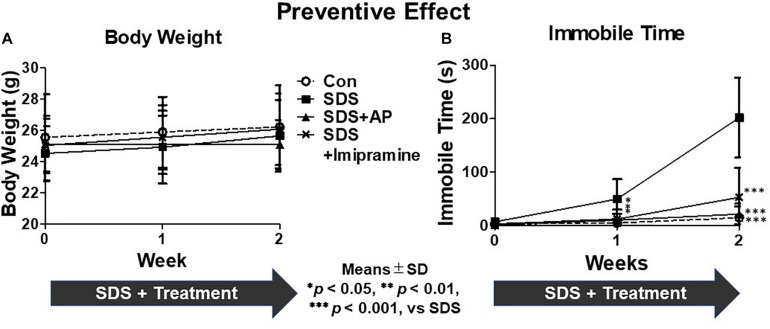
The development of depression symptoms in mice induced by SDS were suppressed by acupuncture. **(A)** Weight changes and **(B)** immobile time in the forced swimming test in mice with SDS stress. Values are means ± SD (*n* = 8). ^∗^*p* < 0.05, ^∗∗^*p* < 0.01, and ^∗∗∗^*p* < 0.001 versus SDS group. AP, acupuncture; SD, standard deviation; SDS, social defeat stress.

First, we investigated the preventive effects of acupuncture in suppressing the development of immobility induced by SDS. There was no significant difference in body weight between the treatment groups during the 2-week SDS induction [*F*(3, 28) = 0.48915, *p* = 0.6926] (Figure 4A). In the FST, we measured whether mice exposed to SDS entered a state of immobility. The immobile time of animals with immobility tended to increase due to stress. In the SDS group without treatment, the immobile time increased significantly [*F*(3, 28) = 17.83603, *p* < 0.0001] (Control vs. SDS, *p* < 0.001) (Figure 4B). Simultaneous acupuncture stimulation of GV20 and Ex-HN3 significantly reduced SDS-induced immobility time (SDS vs. SDS+AP, *p* < 0.001) as well as imipramine treatment (SDS vs. SDS+ imipramine, *p* < 0.001).

**FIGURE 5:**
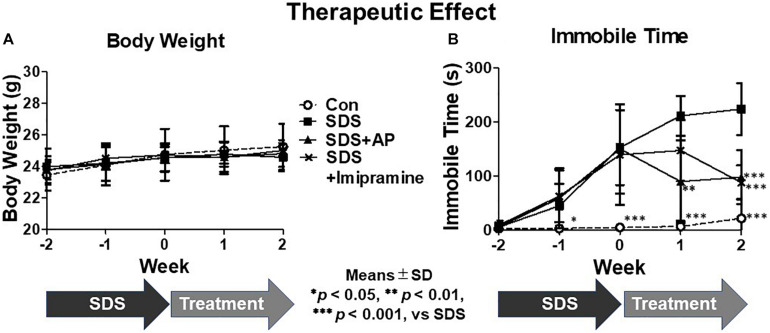
Acupuncture showed therapeutic effect on depressive symptoms in mice induced by SDS. **(A)** Weight changes and **(B)** immobile time in the forced swimming test in mice during treatment of depression induced by SDS stress. Values are means ± SD (*n* = 8). ^∗^*p* < 0.05, ^∗∗^*p* < 0.01, and ^∗∗∗^*p* < 0.001 versus SDS group. AP, acupuncture; SD, standard deviation; SDS, social defeat stress.

In addition, we examined the therapeutic effects of acupuncture on immobility induced by SDS. After stopping exposure to SDS, the immobile time of mice increased for 2 weeks [*F*(3, 28) = 32.15338, *p* < 0.0001] (Control vs. SDS, *p* < 0.001). Simultaneous acupuncture stimulation of GV20 and Ex-HN3 significantly reduced SDS-induced immobility time (SDS vs. SDS+AP, *p* < 0.001) as well as imipramine treatment (SDS vs. SDS+ imipramine, *p* < 0.001) (Figure 5B). There was no significant difference in body weight between the groups [*F*(3, 28) = 0.6794440, *p* = 0.5720] (Figure 5A).

### mRNA Expressions of Neurotrophic Factors

**FIGURE 6:**
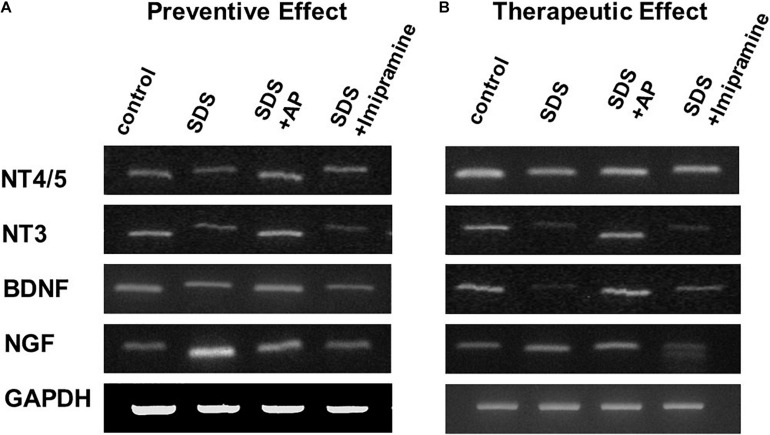
mRNA expressions of neurotrophic factors in the brain of SDS mice. **(A)** mRNA expressions of neurotrophic factors in the brain of SDS mice with ongoing symptoms of depression. **(B)** mRNA expressions of neurotrophic factors in the brain after treatment of depression in SDS mice. AP, acupuncture; BDNF, brain-derived neurotrophic factor; GAPDH, glyceraldehyde 3-phosphate dehydrogenase; NGF, nerve growth factor; NT, neurotrophin; SD, standard deviation; SDS, social defeat stress.

**FIGURE 7:**
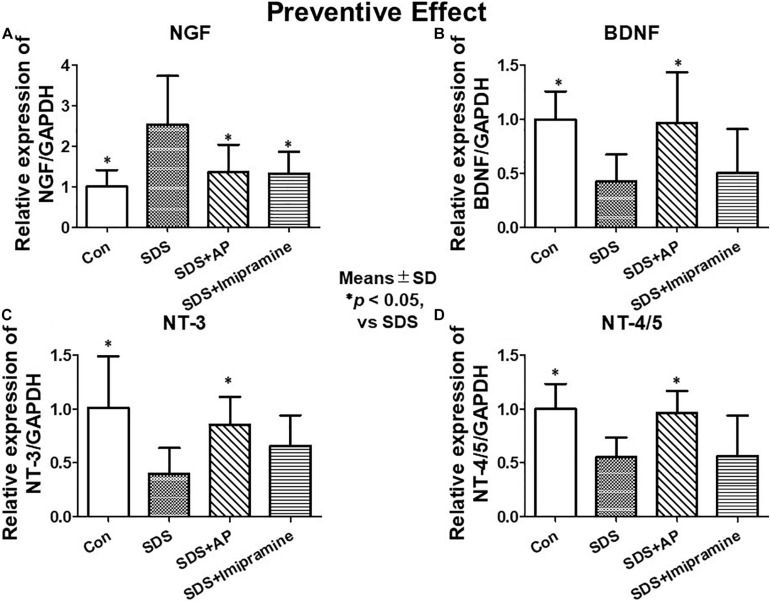
mRNA expressions of neurotrophic factors in the brain of SDS mice with ongoing symptoms of depression quantified by real-time PCR. **(A)** NGF, **(B)** BDNF, **(C)** NT-3, **(D)** NT-4/5. Results are expressed as relative gene expression levels of mRNA. Values are means ± SD (*n* = 8). ^∗^*p* < 0.05 versus SDS group. AP, acupuncture; BDNF, brain-derived neurotrophic factor; GAPDH, glyceraldehyde 3-phosphate dehydrogenase; NGF, nerve growth factor; NT, neurotrophin; SD, standard deviation; SDS, social defeat stress.

To analyze the preventive effects of acupuncture on immobility induced by SDS, we examined the mRNA expression of neurotrophic factors in the brains of SDS mice with ongoing symptoms of depression by RT-PCR and real-time PCR. In the SDS group, mRNA expression of NGF was higher than that in the control group [*F*(3, 28) = 6.113497, *p* = 0.0025] (Control vs. SDS, *p* < 0.05). In the SDS+AP and SDS+ imipramine groups, the mRNA expression of NGF was lower than that in the SDS group (SDS vs. SDS+AP, *p* < 0.05) (SDS vs. SDS+Imipramine, *p* < 0.05) (Figures 6A, 7A). Contrary to NGF expression, mRNA expression of BDNF [*F*(3, 28) = 5.740770, *p* = 0.0034] (Control vs. SDS, *p* < 0.05), NT-3 [*F*(3, 28) = 5.169125, *p* = 0.0057] (Control vs. SDS, *p* < 0.05), and NT-4/5 [*F*(3, 28) = 7.157457, *p* = 0.0010] (Control vs. SDS, *p* < 0.05) were significantly lower than those of the control in the brain of SDS mice. In the SDS+AP group, SDS-reduced BDNF (SDS vs. SDS+AP, *p* < 0.05), NT-3 (SDS vs. SDS+AP, *p* < 0.05), and NT-4/5 (SDS vs. SDS+AP, *p* < 0.05) mRNA expression were restored but not in the SDS+ imipramine group (Figures 6A, 7B,C,D).

**FIGURE 8:**
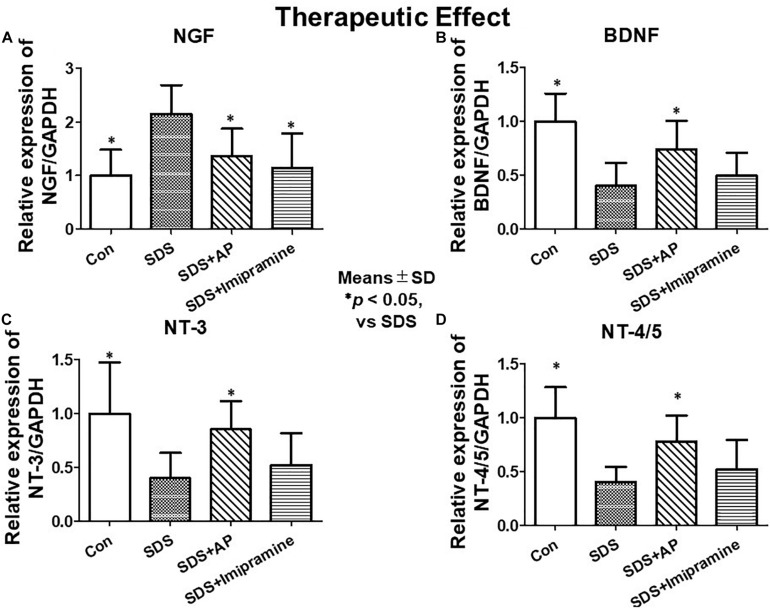
mRNA expressions of neurotrophic factors in the brain after treatment of depression in SDS mice quantified by real-time PCR. **(A)** NGF, **(B)** BDNF, **(C)** NT-3, **(D)** NT-4/5. Results are expressed as relative gene expression levels of mRNA. Values are means ± SD (*n* = 8). ^∗^*p* < 0.05 versus SDS group. AP, acupuncture, BDNF, brain-derived neurotrophic factor; GAPDH, glyceraldehyde 3-phosphate dehydrogenase; NGF, Nerve growth factor; NT, Neurotrophin; SD, standard deviation; SDS, social defeat stress.

To analyze the therapeutic effects, we examined mRNA expression of neurotrophic factors in the brain of SDS mice, which were treated for 2 weeks after completion of SDS, by RT-PCR and real-time RT-PCR. As with the study of preventive effects, NGF was significantly enhanced in the brain of SDS mice [*F*(3, 28) = 7.016273, *p* = 0.0012] (Control vs. SDS, *p* < 0.05). BDNF [*F*(3, 28) = 10.01787, *p* = 0.0001] (Control vs. SDS, *p* < 0.05), NT-3 [*F*(3, 28) = 5.741542, *p* = 0.0034] (Control vs. SDS, *p* < 0.05), and NT-4/5 [*F*(3, 28) = 9.966482, *p* = 0.0001] (control vs. SDS, *p* < 0.05) were significantly suppressed even 2 weeks after the end of SDS (Figures 6B, 8).

SDS-reduced mRNA expression of BDNF (SDS vs. SDS+AP, *p* < 0.05), NT-3 (SDS vs. SDS+AP, *p* < 0.05), and NT-4/5 (SDS vs. SDS+AP, *p* < 0.05) were significantly enhanced by acupuncture but not by imipramine (Figures 6B, 8B,C,D). Enhanced mRNA expression of NGF by SDS was reduced by acupuncture (SDS vs. SDS+AP, *p* < 0.05) and imipramine (SDS vs. SDS+Imipramine, *p* < 0.05) treatment (Figures 6B, 8A).

### Protein Expressions of Neurotrophic Factors

**FIGURE 9:**
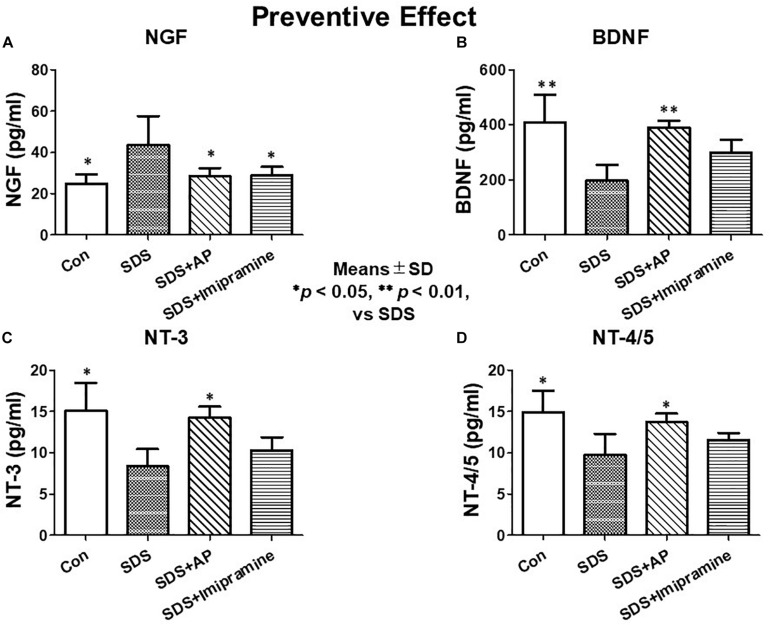
Expressions of neurotrophic factors in the brain of SDS mice with ongoing symptoms of depression. **(A)** NGF, **(B)** BDNF, **(C)** NT-3, **(D)** NT-4/5. Results are expressed as protein content measured by ELISA. Values are means ± SD (*n* = 8). ^∗^*p* < 0.05 and ^∗∗^*p* < 0.01 versus SDS group. AP, acupuncture; BDNF, brain-derived neurotrophic factor; NGF, nerve growth factor; NT, neurotrophin, SD, standard deviation; SDS, social defeat stress.

Protein expression of neurotrophic factors was observed in the same way as mRNA expression by ELISA. In the brain of SDS mice with ongoing symptoms of depression, NGF protein levels were significantly enhanced [*F*(3, 28) = 4.619710, *p* = 0.0095] (Control vs. SDS, *p* < 0.01), and BDNF [*F*(3, 28) = 8.565, *p* = 0.0003] (Control vs. SDS, *p* < 0.01), NT-3 [*F*(3, 28) = 6.331818, *p* = 0.0020] (Control vs. SDS, *p* < 0.01), and NT-4/5 [*F*(3, 28) = 6.155, *p* = 0.0024] (Control vs. SDS, *p* < 0.01) levels were significantly suppressed (Figure 9). The reduced protein levels of BDNF (SDS vs. SDS+AP, *p* < 0.01), NT-3 (SDS vs. SDS+AP, *p* < 0.05), and NT-4/5 (SDS vs. SDS+AP, *p* < 0.05) by SDS were significantly enhanced by acupuncture but not by imipramine (Figures 9B,C,D). SDS-enhanced NGF protein was suppressed by acupuncture (SDS vs. SDS+AP, *p* < 0.05) and imipramine (SDS vs. SDS+Imipramine, *p* < 0.05) (Figure 9A).

**FIGURE 10:**
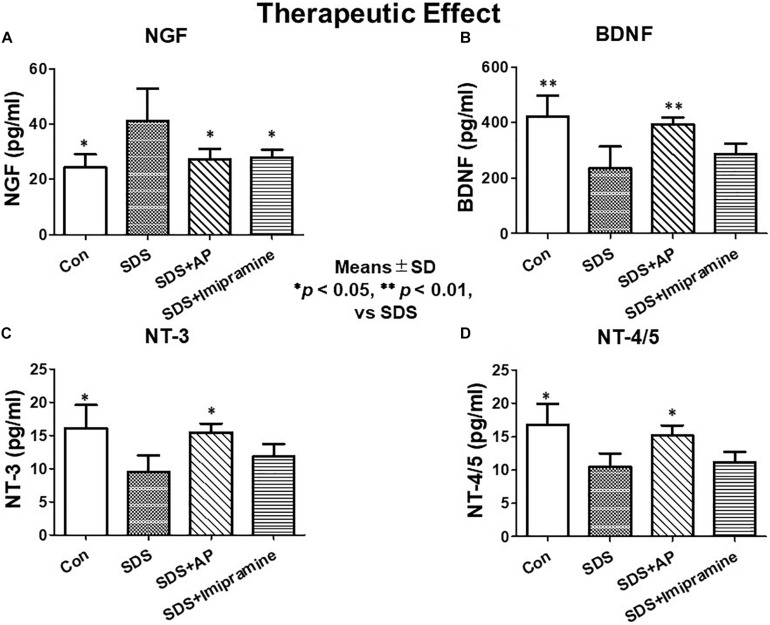
Expressions of neurotrophic factors in the brain after treatment of depression in SDS mice. **(A)** NGF, **(B)** BDNF, **(C)** NT-3, **(D)** NT-4/5. Results are expressed as protein content measured by ELISA. Values are means ± SD (*n* = 8). ^∗^*p* < 0.05 and ^∗∗^*p* < 0.01 versus SDS group. AP, acupuncture; BDNF, brain-derived neurotrophic factor; NGF, nerve growth factor; NT, neurotrophin; SD, standard deviation; SDS, social defeat stress.

In the investigation of therapeutic effects, we examined the protein levels of neurotrophic factors in the brains of SDS mice treated for 2 weeks after completion of SDS. As with mRNA expression analysis, NGF protein was significantly enhanced [*F*(3, 28) = 5.583477, *p* = 0.0039] (Control vs. SDS, *p* < 0.05) in the brains of mice 2 weeks after the end of SDS. Protein levels of BDNF [*F*(3, 28) = 9.429835, *p* = 0.0002] (Control vs. SDS, *p* < 0.01), NT-3 [*F*(3, 28) = 5.188506, *p* = 0.0056] (Control vs. SDS, *p* < 0.05), and NT-4/5 [*F*(3, 28) = 5.890445, *p* = 0.0030] (Control vs. SDS, *p* < 0.05) were significantly suppressed. SDS-reduced protein levels of BDNF (SDS vs. SDS+AP, *p* < 0.01), NT-3 (SDS vs. SDS+AP, *p* < 0.05), and NT-4/5 (SDS vs. SDS+AP, *p* < 0.05) were significantly enhanced by acupuncture but not by imipramine (Figures 10B,C,D). Similar to the study investigating preventive effects, acupuncture and imipramine suppressed SDS-enhanced NGF protein (SDS vs. SDS+AP, *p* < 0.05) (Figure 10A).

## Discussion

Many animal studies have examined the state of depression (Cryan et al., 2005; Petit-Demouliere et al., 2005; Tsankova et al., 2006). Some researchers have demonstrated the effects of electroacupuncture at the GV20 “Bai-Hui” and Ex-HN3 “Yintáng” points, GV20, and Sp-6 “Sanyinjiao” points, and GV20 and EX17 “An-Mian” points in depression animal models (Liu et al., 2007; Zhu et al., 2009; You et al., 2010). We have reported that simultaneous acupuncture stimulation as part of GV20 and Ex-HN3 improves the symptoms of a rat model of water-immersion-induced depression (Tanahashi et al., 2016; Takagi et al., 2017) and a mouse model of FS-induced depression (Yamamoto et al., 2018).

For many acupuncture researchers, setting a control stimulus is a very challenging problem in experiments. In addition, it is also necessary to consider whether sham needles can be used as a control. There are several types of sham needles available today. However, we were not able to obtain any sham needles applicable to animals that could be used as a control for horizontal subcutaneous acupuncture stimulation of the head as in this study. This is because stimulation of a different part of the body than the target acupuncture point usually has an effect on the body and is not suitable as a control. For these reasons, we set up a group with no acupuncture as a control in this series of studies. Takagi et al. (2017) showed that acupuncture with either GV20 or EX-HN3 alone was not effective in suppressing immobility in water-immersion stressed rats. However, there are few studies on the single use of acupuncture points that show efficacy in combinations of two or more. Additional studies will be required to assess the contribution of specific locations, number of points, subdermal stimulation or other inflammatory factors in the effects of acupuncture.

In this study, we examined the effect of simultaneous acupuncture stimulation as part of GV20 and Ex-HN3 using SDS-treated mice that has been developed as a new ethological model of depression that more closely models the psychological stress that humans can experience during antagonistic social interactions. The experiments were performed in two parts. The first was to verify the effect of acupuncture on preventing SDS-induced immobility, and the second was to verify the effect of acupuncture on SDS-induced immobility that had already developed. 2 weeks of SDS significantly enhanced the immobile time of mice, that is, induced depressive-like symptoms (Figure 4B). Simultaneous acupuncture stimulation, as part of GV20 and Ex-HN3, prevented immobility as well as antidepressants. After the end of SDS, an increase in the immobile time of mice was also observed even 2 weeks later (Figure 5B), revealing that the effects of SDS persisted long after the end of SDS (Figure 5B). Simultaneous acupuncture stimulation as part of GV20 and Ex-HN3 showed a therapeutic effect as well as a suppressive effect on SDS-induced immobility. Previously studies indicated that acupuncture is effective in the neurological system not only as a tool to treat disease, but also as a disease preventive strategy (Li and Wang, 2013). The results of our study showed that simultaneous acupuncture stimulation of GV20 and EX-HN3 can be used not only as a treatment for depression that has already developed, but also as a routine maintenance to avoid depression.

Various theories have been proposed as the causes of depression. The oldest hypothesis is the “monoamine hypothesis,” which states that depression may be caused by decreased levels of monoamines in the brain (Nestler et al., 2002; Liu et al., 2007; Schlaepfer et al., 2010). It is also known that acupuncture stimulates monoamines in the brain (Xu et al., 2007; Murotani et al., 2010). Recently, it has been proposed that decreased neurotrophic factor production, increased glutamate-induced intracellular calcium concentration, and abnormal intracellular signal transduction mechanisms related to neuroplasticity by cortisol via the hypothalamic-pituitary-adrenocortical (HPA) axis are also thought to be the cause of depression (Jacobson and Sapolsky, 1991; Nibuya et al., 1995; Manji et al., 2001; Sanacora et al., 2008). In our previous study, simultaneous acupuncture stimulation of GV20 and Ex-HN3 was shown to reduce blood corticosterone levels in depressed rats (Tanahashi et al., 2016).

In this study, we focused on the expression of neurotrophic factors in the brain, which are key components of depression. To examine the mechanism of preventive and therapeutic effects of acupuncture on depression, we examined the expression of neurotrophic factors in the brains of SDS mice.

The relationship between stress that causes depression and neurotrophic factors has been identified. Stress has been reported to reduce the expression of BDNF mRNA in the hippocampus (Nibuya et al., 1995; Smith et al., 1995). It has also been reported that electroconvulsive stimulation and chronic administration of antidepressants improve the reduction of BDNF mRNA in the rat hippocampus due to stress (Nibuya et al., 1995). Furthermore, chronic administration of tricyclic antidepressants has been reported to increase BDNF and NT-3 protein levels in rodent brains (Okamoto et al., 2003; Tsankova et al., 2006). NT-3 and NT-4/5 are neurotrophic factors that are structurally related to NGF and BDNF. These neurotrophic factors promote the growth and survival of nerve cells, such as BDNF (Barde, 1994; Lindholm, 1994; Zheng et al., 1995; Pérez-Navarro et al., 2000). Unlike NGF and BDNF, there are not many studies currently examining NT-3 and NT-4/5 in depression. Otsuki et al. (2008) examined the expression of NGF, BDNF, NT-3, and NT-4/5 mRNAs in peripheral blood cells of patients with depression and bipolar disorder. They reported that the expression level of NT-3 mRNA decreased in a symptom-dependent manner in these diseases. NT-3 is thought to play a role in the neurobiological processes associated with mood and anxiety disorders. In recent years, it has been proposed that NT-3 is a potential pharmacological target for mood disorders due to its effects on monoamine neurotransmitters, regulation of synaptic plasticity and neurogenesis, enhancement of BDNF signaling, and regulation of the HPA axis (de Miranda et al., 2020). There are even fewer studies on the role of NT-4/5 in depression. Liu et al. (2017) revealed that intranasal administration of recombinant adeno-associated virus (AAV) expressing NT4/5-NAP (Asn-Ala-Pro-Val-Ser-Ile-Pro-Gln, NAPVSIPQ) exerts antidepressant effects in socially isolated mice.

Simultaneous acupuncture stimulation as part of GV20 and Ex-HN3 restored the expression of SDS-reduced BDNF, NT-3, and NT-4/5. However, imipramine administration suppressed SDS-induced immobility but did not restore these neurotrophic factors. The discrepancy with previous studies is attributed to the effects of treatments on neurotrophic factor recoveries seen in 2 weeks with acupuncture but more time with antidepressants. In addition, differences in animal species or stress types may also be the cause of this discrepancy.

In contrast to neurotrophic factors, such as BDNF, NGF expression was increased in SDS mice. Contrary to our results in SDS, some studies have reported reduced NGF expression in depression (Okamoto et al., 2003). However, increased NGF expression after depressive stress has also been reported (Hadjiconstantinou et al., 2001; de Azevedo Cardoso et al., 2014). Acupuncture stimulation and imipramine suppressed the increase in NGF expression induced by SDS. The reason that shows the behavior only the opposite NGF of neurotrophic factor expression in SDS mouse is still unclear.

As described above, improvements in the production of monoamine and serotonin, the HPA axis, and expression of neurotrophic factors have been proposed as treatments for depression (Jacobson and Sapolsky, 1991; Smith et al., 1995; Manji et al., 2001; Nestler et al., 2002; Okamoto et al., 2003; Adams et al., 2008; Otsuki et al., 2008; Sanacora et al., 2008; Liu et al., 2017; de Miranda et al., 2020). Our studies revealed that acupuncture stimulation ameliorates SDS-induced immobility in mice. The treatment mechanism of acupuncture may be different from that of antidepressants because its effects on neurotrophic factors appear earlier. The therapeutic effects on neurological disorders that have been studied so far have been shown to be related to the mediation of neuroplasticity and regulation of neurotrophic factors and neurotransmitters, but the exact mechanism underlying the effects of acupuncture has not been clarified yet (Xiao et al., 2018). In this study using our SDS-treated mice, it is clarified that simultaneous acupuncture stimulation as part of GV20 and Ex-HN3 strongly restored the expression of neurotrophic factors altered by immobility to their respective normal states, suggesting that this might be a promising therapeutic strategy for depressive disorder.

## Data Availability Statement

The raw data supporting the conclusions of this article will be made available by the authors, without undue reservation.

## Ethics Statement

The animal study was reviewed and approved by Animal Experiment Ethical Review Committee of Suzuka University of Medical Science (Permission number: 189).

## Author Contributions

JK, TI, NT, and NM contributed to the conception and design of the study. JK, KT, TY, and NN conducted experiments and organized a database. JK performed the statistical analysis and wrote draft of the manuscript. All authors contributed to manuscript revision, read, and approved the submitted version.

## Conflict of Interest

The authors declare that the research was conducted in the absence of any commercial or financial relationships that could be construed as a potential conflict of interest.

## Publisher’s Note

All claims expressed in this article are solely those of the authors and do not necessarily represent those of their affiliated organizations, or those of the publisher, the editors and the reviewers. Any product that may be evaluated in this article, or claim that may be made by its manufacturer, is not guaranteed or endorsed by the publisher.
