# Supplementary material for: Acupuncture Treatment for Social Defeat Stress
Source: Front Behav Neurosci. 2021 Jul 28;15:685433. doi: 10.3389/fnbeh.2021.685433 (PMC8355549; doi:10.3389/fnbeh.2021.685433)
Supplement: Supplementary file 1 [file Data_Sheet_1.PDF]

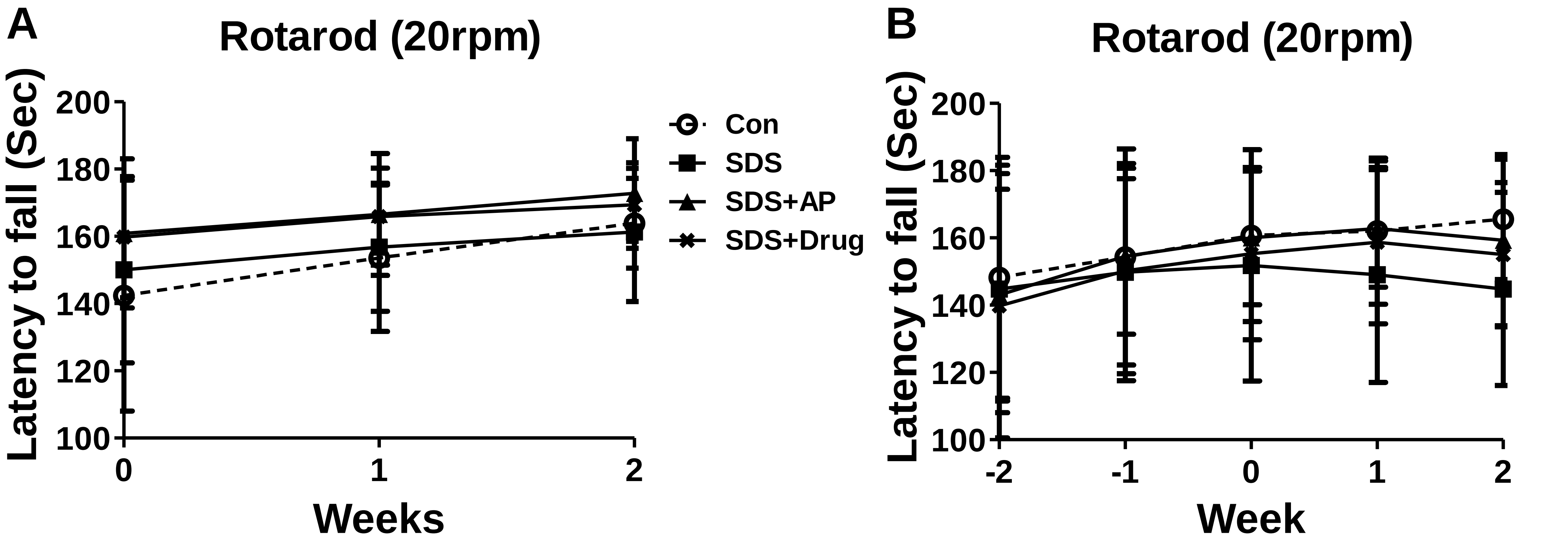

Supplemental figure1. Effect of SDS on locomotor performance of mice. Rotarods at 20 rpm were performed for 180 seconds and the time to fall of the mouse was measured. (A) Latency to fall of SDS mice with ongoing symptoms of depression. (B) Latency to fall of SDS mice after treatment of depression. Values are means ± SD (n = 6). There were no significant differences between all groups. AP: Acupuncture, SD: Standard deviation, SDS: Social defeat stress.
